# Supplementary material for: Mimicked synthetic ribosomal protein complex for benchmarking crosslinking mass spectrometry workflows
Source: Nat Commun. 2022 Jul 8;13:3975. doi: 10.1038/s41467-022-31701-w (PMC9270371; doi:10.1038/s41467-022-31701-w)
Supplement: Supplementary file 3 — Description of Additional Supplementary Files [file 41467_2022_31701_MOESM3_ESM.docx]

**Legends for Supplementary Files**

Supplementary Data 1: List of all synthesized peptides and their annotation to groups for crosslinking

Supplementary Data 2: List of all crosslink IDs at 1% estimated FDR from crosslinked library samples measured without FAIMS and experimentally validated FDRs from main, enrichable and acidic library.

Supplementary Data 3: Search settings used for MeroX, Annika, XlinkX, pLink 2, MaxLynx and xiSearch analyses.
