# Supplementary material for: Mimicked synthetic ribosomal protein complex for benchmarking crosslinking mass spectrometry workflows
Source: Nat Commun. 2022 Jul 8;13:3975. doi: 10.1038/s41467-022-31701-w (PMC9270371; doi:10.1038/s41467-022-31701-w)
Supplement: Supplementary file 7 — Reporting Summary [file 41467_2022_31701_MOESM7_ESM.pdf]

nature portfolio

Corresponding author(s): Karl Mechtler, Manuel Matzinger

Last updated by author(s): Apr 14, 2022

Reporting Summary

Nature Portfolio wishes to improve the reproducibility of the work that we publish. This form provides structure for consistency and transparency in reporting. For further information on Nature Portfolio policies, see our Editorial Policies and the Editorial Policy Checklist.

Statistics

For all statistical analyses, confirm that the following items are present in the figure legend, table legend, main text, or Methods section.

n/a	Confirmed	
☐	☒	The exact sample size (*n*) for each experimental group/condition, given as a discrete number and unit of measurement
☐	☒	A statement on whether measurements were taken from distinct samples or whether the same sample was measured repeatedly
☐	☒	The statistical test(s) used AND whether they are one- or two-sided<br>*Only common tests should be described solely by name; describe more complex techniques in the Methods section.*
☒	☐	A description of all covariates tested
☒	☐	A description of any assumptions or corrections, such as tests of normality and adjustment for multiple comparisons
☐	☒	A full description of the statistical parameters including central tendency (e.g. means) or other basic estimates (e.g. regression coefficient) AND variation (e.g. standard deviation) or associated estimates of uncertainty (e.g. confidence intervals)
☐	☒	For null hypothesis testing, the test statistic (e.g. *F*, *t*, *r*) with confidence intervals, effect sizes, degrees of freedom and *P* value noted<br>*Give P values as exact values whenever suitable.*
☒	☐	For Bayesian analysis, information on the choice of priors and Markov chain Monte Carlo settings
☒	☐	For hierarchical and complex designs, identification of the appropriate level for tests and full reporting of outcomes
☒	☐	Estimates of effect sizes (e.g. Cohen's *d*, Pearson's *r*), indicating how they were calculated

Our web collection on statistics for biologists contains articles on many of the points above.

Software and code

Policy information about availability of computer code

Data collection	Mass spectrometers used for data acquisition were operated using Thermo Scientific Xcalibur v4.2.4.7 (HF-X devices) or v 4.4.16.14 (Exploris devices)
Data analysis	For Data analysis the following tools were used: MeroX standalone/ v 2.0.1.4; MS Annika in PD 2.5, v 1.2.17259; XlinkX integrated to PD 2.5; pLink standalone/ v2.3.9; xiSearch/xiFDR standalone xi1.7.6.5/xiFDR2.1.5.5; MaxLynx integrated to MaxQuant v 2.0.2.0.<br>Post processing was done via IMP-X-FDR that using the following modules: Biopython 1.79 (using the submodules Bio package, Bio.SeqUtils subpackage and Bio.SeqUtils.ProtParam), matplotlib_venn 0.11.6, openpyxl 3.0.9, toolz 0.11.2, venn 0.1.3, xlrd 1.2.0, XlsxWriter 3.0.3 and seqlogo 5.29.843. Custom code for IMP-X-FDR is availible on github (https://github.com/fstanek/imp-x-fdr)

For manuscripts utilizing custom algorithms or software that are central to the research but not yet described in published literature, software must be made available to editors and reviewers. We strongly encourage code deposition in a community repository (e.g. GitHub). See the Nature Portfolio guidelines for submitting code & software for further information.

Data

Policy information about availability of data

All manuscripts must include a data availability statement. This statement should provide the following information, where applicable:

- Accession codes, unique identifiers, or web links for publicly available datasets
- A description of any restrictions on data availability
- For clinical datasets or third party data, please ensure that the statement adheres to our policy

Raw data and result files are accessible via the PRIDE repository using the identifier PXD029252

Field-specific reporting

Please select the one below that is the best fit for your research. If you are not sure, read the appropriate sections before making your selection.

☒ Life sciences ☐ Behavioural & social sciences ☐ Ecological, evolutionary & environmental sciences

For a reference copy of the document with all sections, see nature.com/documents/nr-reporting-summary-flat.pdf

Life sciences study design

All studies must disclose on these points even when the disclosure is negative.

Sample size	Data was recorded in tripilicates measured on different days using the same settings by mass spectrometry. For the main library we used 20 groups of 6-10 synthetic peptides from which the theoretical maximum number of crosslinks that can be formed is 1018. For the enrichable library we used 16 groups of 7-8 synthetic peptides from which the theoretical maximum number of crosslinks that can be formed is 512. For the acidic library we used 13 groups of 6-7 synthetic peptides from which the theoretical maximum number of crosslinks that can be formed is 280.<br>Besides that, no calculations for sample size have been done as no biological variation is to be expected in this synthetic peptide library set.
Data exclusions	No data were excluded from the analysis.
Replication	Reproducability by means of ID numbers was estimated by calculation of standard deviations. Reproducability by means of Id type was visualized by creating Venn diagrams, visualized in the mansucript figures.
Randomization	Not applicable to the study, as this is a synthetic peptide library and we do not investigate any biological effects in this study. The same library was used for the whole study.
Blinding	As this is not a clinical study, blinding is not applicable.

Reporting for specific materials, systems and methods

We require information from authors about some types of materials, experimental systems and methods used in many studies. Here, indicate whether each material, system or method listed is relevant to your study. If you are not sure if a list item applies to your research, read the appropriate section before selecting a response.

Materials & experimental systems

n/a	Involved in the study	
☒	☐	Antibodies
☐	☒	Eukaryotic cell lines
☒	☐	Palaeontology and archaeology
☒	☐	Animals and other organisms
☒	☐	Human research participants
☒	☐	Clinical data
☒	☐	Dual use research of concern

Methods

n/a	Involved in the study	
☒	☐	ChIP-seq
☒	☐	Flow cytometry
☒	☐	MRI-based neuroimaging

Eukaryotic cell lines

Policy information about cell lines

Cell line source(s)	HEK293T Lenti-X cells (TaKaRa) were used only as to mimick a complex matrix in samples of crosslinked peptide library.
Authentication	Cell line was purchased directly from provider (TaKaRa bio, Cat# 632180), expanded and frozen at passage 4. Early passages of this stock collection were<br>used for all experiments. Cell line was not further authenticate
Mycoplasma contamination	Cell lines were not regulary tested for mycoplasma status.
Commonly misidentified lines<br>(See ICLAC register)	No commonly misidentified cell lines were used in the study.
